# Histological and biomechanical properties of systemic arteries in young and old Warmblood horses

**DOI:** 10.1371/journal.pone.0253730

**Published:** 2021-07-12

**Authors:** Lisse Vera, Sofie Muylle, Glenn Van Steenkiste, Patrick Segers, Annelies Decloedt, Koen Chiers, Gunther van Loon

**Affiliations:** 1 Department of Large Animal Internal Medicine, Faculty of Veterinary Medicine, Equine Cardioteam Ghent University, Ghent University, Merelbeke, Belgium; 2 Department of Morphology, Faculty of Veterinary Medicine, Ghent University, Merelbeke, Belgium; 3 IBiTech-bioMMeda, Ghent University, Ghent, Belgium; 4 Department of Pathology, Bacteriology and Poultry Diseases, Faculty of Veterinary Medicine, Ghent University, Merelbeke, Belgium; University of Zurich, SWITZERLAND

## Abstract

Arterial rupture is a well-recognized cause of sudden death in horses, which mainly affects older horses. The arterial wall is known to stiffen with age, although the underlying age-related histological and biomechanical changes remain unclear. The purpose of this study was to investigate the effect of aging by histological analysis of the arterial wall and examination of the arterial wall biomechanical properties using an inflation-extension test. Entire circular samples of the proximal and distal aorta, cranial and caudal common carotid, external iliac, femoral and median artery were collected from 6 young (6 years) and 14 old horses (≥15 years). Samples of all arteries were histologically examined and intima media thickness as well as area % of elastin, smooth muscle actin and collagen type I and III were determined. Older horses had a significantly larger intima media thickness and a significantly higher area % of smooth muscle actin compared to young horses. Samples of the proximal and distal aorta, the caudal common carotid and the external iliac artery were mechanically assessed using an in-house developed inflation-extension device with ultrasound analysis. Rupture occurred in a minority of arteries (8/78) at high pressures (between 250–300 mmHg), and mostly occurred in older horses (7/8). Pressure-area, pressure-compliance and pressure-distensibility curves were constructed. A significant difference in the pressure-area curves of the distal aorta, common carotid artery and external iliac artery, the pressure-compliance curves of the proximal aorta and carotid artery and the pressure-distensibility curve of the proximal aorta was observed between young and old horses. Results demonstrate an effect of age on the histological and biomechanical properties of the arterial wall, which might explain why arterial rupture occurs more often in older horses.

## Introduction

Conduit arteries in mammals are compliant by nature and provide a low resistance path for the blood supply to the visceral organs and the limbs. At the same time, they cushion the pulsatile action of the heart and keep systolic and diastolic pressure within physiological limits [1]. The key structural components contributing to the compliance of the arterial wall are elastin fibres, collagen fibres, smooth muscle cells and cross-linking matrix constituents [1, 2]. In humans, luminal enlargement and arterial wall thickening of the conduit arteries are known to occur with increasing age, resulting in arterial wall stiffening [3, 4]. The arterial compliance at physiological pressures and the pressure at which compliance is maximal decrease with age, indicating a shift of the pressure-compliance curve to lower pressures [5]. These mechanical alterations are due to major structural changes. Structural changes are characterised by a proinflammatory profile, collagen deposition and fragmentation and thinning of elastin fibres [1, 3, 6, 7]. The elastin fragmentation causes luminal enlargement [8] and leads to a transfer of part of the mechanical load to the collagen fibres, which are 100 to 1000 times stiffer compared to elastin fibres [1]. Due to calcification [1, 3, 6] and the accumulation of advanced glycation end products (AGEs) [1, 3] the elastin fibres become stiffer. The formation of AGEs is not limited to elastin fibres but also occurs in collagen fibres [1]. In the aged vascular wall, the intima is infiltrated by vascular smooth muscle cells from the adjacent tunica media. Vascular smooth muscle cells, having switched from the contractile to the synthetic phenotype, are capable of migration towards the intima. Once in the tunica intima they start to proliferate and synthesise extracellular matrix [9–11], causing thickening of the arterial wall [1]. Aging not only causes thickening of the tunica intima but also increases tunica media thickness, while its cellularity decreases due to vascular smooth muscle cell hypertrophy and the build-up of extracellular matrix [12].

In horses, little is known about the alterations in vascular properties due to aging. Nevertheless, arterial rupture is known to occur more often in older horses, especially associated with parturition in mares [13], coitus in stallions [14], intense exercise [15] or the administration of α1-agonists for treatment of left dorsal displacement of the large colon [16]. Recently, a non-invasive ultrasound study demonstrated stiffening of the conduit arteries in combination with luminal enlargement and arterial wall thickening with age in horses [17]. This might imply that structural changes of the arterial wall due to aging are likely to be one of the contributing factors leading to arterial rupture. The objective of the current study was to determine whether age-related functional changes found in the *in vivo* study in horses can be supported by structural and mechanical differences, using histology and an *ex vivo* inflation-extension test with ultrasound analysis.

## Materials and methods

### Sample collection and preparation

**Fig 1:**
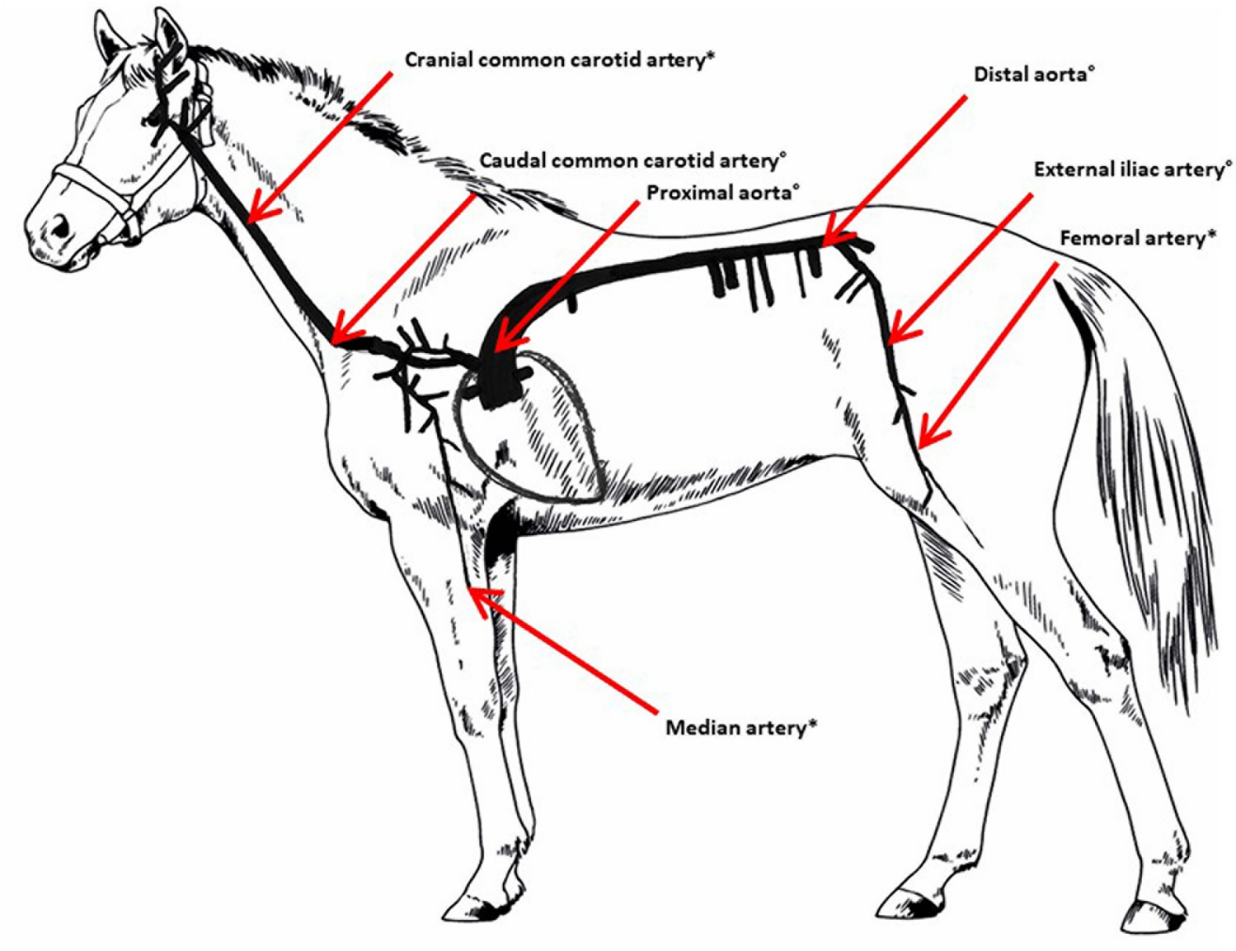
Schematic overview of the locations at which samples were collected. *: samples of 14 old and 6 young horses were collected for histology. °: samples of 14 old and 6 young horses were collected for histology and biomechanical testing.

Arterial tissue was collected from old (≥15 years) and young (6 years) Warmblood horses, euthanized for non-cardiovascular reasons. According to the European Directive 2010/63 and the Belgian Royal Decree on the protection of animals used for experimental purposes, as well as the guidelines of the Local Ethical Committee of the Faculty of Veterinary Medicine, Ghent University, the use of tissue from animals euthanized for clinical reasons did not require approval by the committee. For each horse, 10 cm long cylindrical tissue samples from the entire arterial wall were collected at seven locations. The left common carotid artery was sampled at the level of the thoracic inlet (caudal common carotid artery) and 30 cm more cranially (cranial common carotid artery). The aorta was sampled just distal to the sinotubular junction (proximal aorta) and just cranial from the bifurcation into the external and internal iliac arteries (distal aorta). The left external iliac artery was sampled proximal to the arteria profunda femoris and the left femoral artery was sampled 20 cm distal to the arteria profunda femoris. The left median artery was sampled proximal to the carpus (Fig 1). All samples were collected within 12 hours post euthanasia. From the proximal and distal aorta, the caudal common carotid and the external iliac artery 5–7 cm of the arterial sample was immediately frozen at -80°C for the *ex vivo* inflation-extension test. The exact in situ length was not recorded prior to sampling. The remaining part of these samples, as well as the samples of the cranial common carotid, femoral and median artery were fixed in buffered 4% formaldehyde solution for 24 hours, routinely embedded in paraffin wax and cut into 5 μm longitudinal and cross-sectional sections for histological examination. Sections were stained with haematoxylin and eosin to evaluate the different layers of the arterial wall. Immunohistochemistry was performed to demonstrate the presence of smooth muscle cells, elastin, collagen type I and collagen type III [18, 19]. Smooth muscle cells were visualised using mouse anti-smooth muscle actin (1/200, DakoCytomation). The presence of elastin was demonstrated using a monoclonal mouse anti-human anti-elastin antibody BA-4 (1/600, Leica Biosystems) in combination with a standard avidin-biotin complex method with diaminobenzidine as chromogen for visualization (Envision, Dako). For both smooth muscle cells and elastin, immunolabeling was achieved using a high-sensitivity horseradish peroxidase diaminobenzidine kit (Envision DAB+ kit, Dako) in an immunostainer (Cytomation S/N S38-7410-01, Dako). Samples were labelled with monoclonal rabbit anti-collagen type I (1/100, ABCAM 138492) and III (1/200, ABCAM 7778). Visualization was obtained using peroxidase labelled polymer conjugated to goat anti-rabbit immunoglobulins (Agilent, K400311-2, Dako) and biotinylated polyclonal goat anti-rabbit immunoglobulins (Agilent, E043201-8, Dako), respectively, in combination with a peroxidase diaminobenzidine kit (DAB+ Liquid, K346811-2, Agilent).

#### Histology

All sections of 140 arterial samples were randomised and analysed blindly. Haematoxylin eosin sections were used to measure the combined thickness of the tunica intima and the tunica media and to screen for arterial wall lesions and mineralisations. A low power magnification was used to measure the intima and media thickness as the perpendicular distance between the endothelial layer and the transition tunica media to tunica adventitia. For each section five measurements were carried out at randomly chosen locations over the entire cross-section of the arterial wall and the mean of those values was calculated. The intimal and medial thickness of the proximal aorta was not assessed, as wall thickness exceeded the measurement capacity. The area % of elastin, smooth muscle actin, collagen type I and collagen type III were determined using image analysis [18, 19]. For each sample the threshold of positive (brown) staining was set manually to assure the detection of all different intensities of positive staining without background. The area % were determined using a Leica Camera DFC320 (Leica Microsystems Ltd) coupled to a computer-based image analysis system LAS v.3.8. (Leica Microsystems Ltd.) at 400x magnification [19]. For each slide, five randomly selected image frames representing the entire thickness from just underneath the endothelium until the transition of tunica media to tunica adventitia were analysed and the mean area % of the five image frames were calculated. In many cases the tunica media consisted of two separate layers. If so, image analysis was performed separately for both layers and the thickness of each layer was measured. To calculate the overall area % of elastin, smooth muscle actin and collagen type I and III, the following formula was applied:

$$overall\;area\;\%=mean\;area\;\%\;in\;layer\;1\;*\left(\frac{mean\;thickness\;of\;layer\;1}{mean\;total\;thickness}\right)+mean\;area\;\%\;in\;layer\;2\;*\left(\frac{mean\;thickness\;of\;layer\;2}{mean\;total\;thickness}\right)$$

#### Inflation-extension test

**Fig 2:**
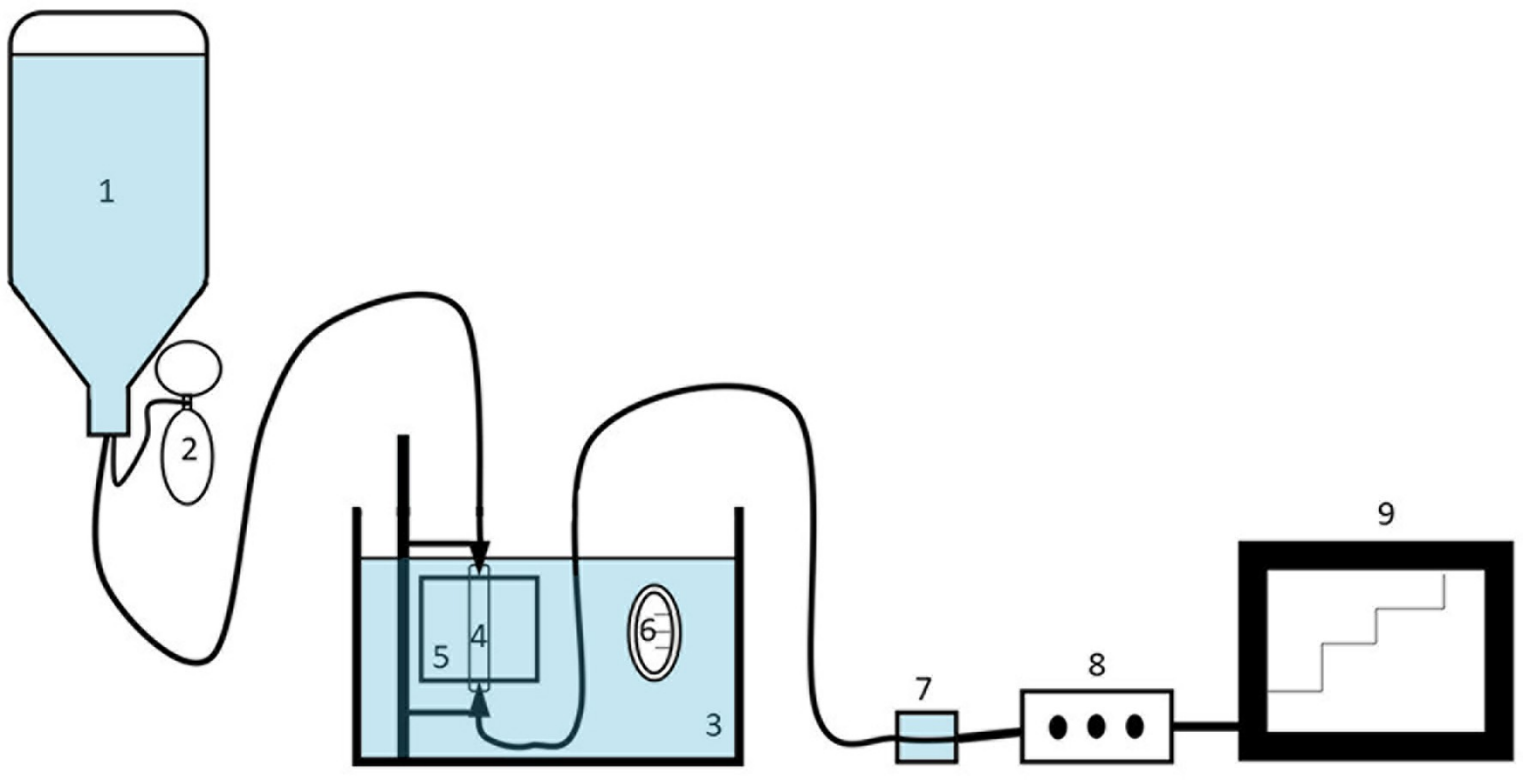
Schematic overview of the custom-made pressurisation system for the inflation-extension test under ultrasound monitoring. 1: pressure reservoir containing 0.9% NaCl at 37°C; 2: inflation balloon to adjust pressure; 3: water bath containing 0.9% NaCl at 37°C; 4: pressurised arterial sample; 5: silicone window in the water tank, through which ultrasound images were obtained; 6: bath thermometer; 7: fluid filled pressure transducer; 8: acquisition station; 9: visualisation of actual pressure in the arterial segment on screen.

**Fig 3:**
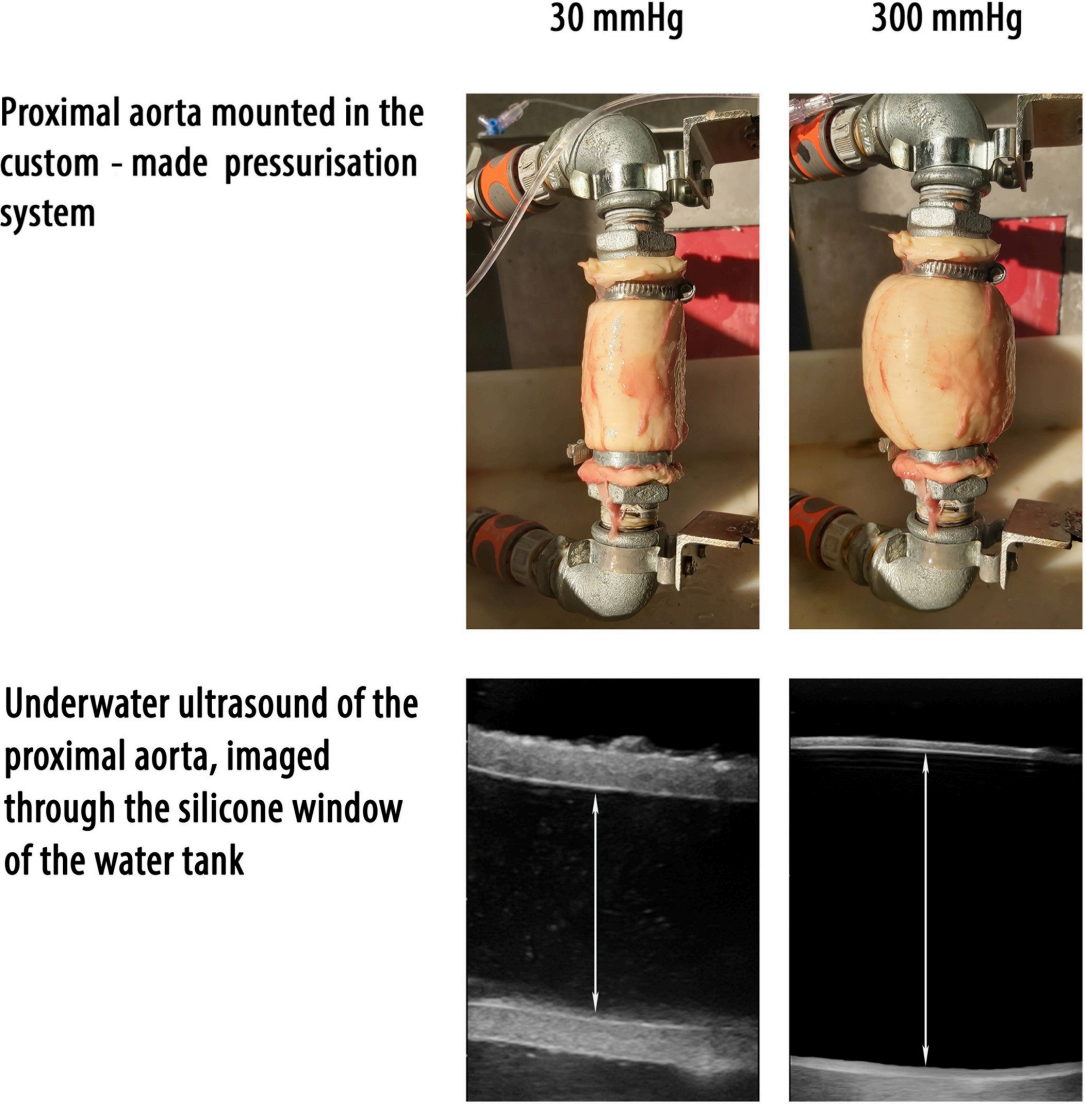
Example of the proximal aorta, mounted in the pressurisation system but out of the water bath, and derived ultrasound images at 30 (left) and 300 (right) mmHg. Double-sided arrows indicate diameter measurements performed from inner edge to inner edge.

For the *ex vivo* inflation-extension test, samples were thawed at room temperature. Loose connective tissue was removed and all side branches were ligated. Subsequently, the vessel was vertically mounted onto a custom-made pressurisation system (Fig 2) with adaptable connectors depending on the size of the arterial segment. Each vessel side was fixed using either two ligatures (common carotid artery and external iliac artery) or a tie strap (proximal and caudal aorta) (Fig 3). Each vessel was first fixed to the connectors. After fixation the vessel was inflated to a pressure of 120 mmHg during which the connectors could freely move in vertical direction. At a pressure of 120 mmHg, the distance between both connectors, and thus the length of the vessel, was fixed and remained the same during the entire experiment. The vessel was placed in a 0.9% NaCl water bath at fixed length at a constant temperature of 37°C. Before starting pressurisation, all vessels were pre-inflated three times to a pressure of 120 mmHg for 1 minute. Afterwards all vessels (n = 78) were pressurised using a pressure reservoir containing 0.9% NaCl at 37°C. The pressure was regulated using an inflation bulb connected to the pressure reservoir. Pressure was increased by inflating air and decreased by releasing fluid. The pressure inside the artery was digitally monitored using a fluid filled pressure transducer (MLT0699 Disposable BP Transducer, ADInstruments) connected to the distal end of the arterial segment. The pressure was visualised using a digital acquisition station (PowerLab 8, ADInstruments). Measurements were sequentially performed at 15, 30, 45, 60, 80, 100, 125, 200, 250 and 300 mmHg. Each pressure point was kept static for 1 minute, during which three B-mode longitudinal ultrasonographic video-loops were collected (Vivid IQ, GE Healthcare) through a silicone rubber window in the water tank, using a 9 MHz linear probe (9L-RS, GE Healthcare). Longitudinal alignment of the probe was ensured by clearly visualising the intima of the arterial wall in both the near and the far field. All loops were digitally stored as raw data (Echopac version 203, GE Healthcare) and blinded for further offline analysis (MicroDicom viewer version 3.2.7). On each video loop the inner arterial diameter was measured from inner edge to inner edge (Fig 3). For each pressure point the mean inner arterial diameter was calculated as the average of three measurements from separately obtained video loops.

### Statistical analysis

#### Histology

Statistical analysis was performed using SPSS version 25. For the histological results normality of all variables was checked graphically using Q-Q plots, in which only minimal deviations of the data from a straight diagonal line indicated normal distribution. A univariate ANOVA was used including horse as random factor and age (young or old) and location (proximal and distal aorta, cranial and caudal common carotid artery, external iliac artery, femoral artery and median artery) as fixed factors to compare the overall thickness and the overall area % of smooth muscle actin, elastin, collagen type I and collagen type III. For location, post-hoc Bonferroni correction for multiple comparisons was applied. P-values ≤0.05 were considered statistically significant.

#### Inflation-extension test

Statistical analysis was performed using R version 3.2.6 and Matlab. For the inflation—extension test, results were fitted to the arctangent model of Langewouters et al. [20] The area for a given pressure was calculated as, **$A(P)=A_{m}\left\{\frac{1}{2}+\frac{1}{\pi }tan^{-1}\left(\frac{P-P_{0}}{P_{1}}\right)\right\}$**, A_m_ represents the maximal cross-sectional area of the investigated artery, P_0_ the pressure at which compliance is maximal and P_1_ the half-width pressure. Corresponding compliance was calculated as $C_{A}(P)=\frac{\frac{A_{m}}{\pi P_{1}}}{1+{\left(\frac{P-P_{0}}{P_{1}}\right)}^{2}}$ and distensibility as **$D_{A}(P)=\frac{C_{A}}{A}$**. [20]. The effect of age (young or old) on the pressure-area, pressure-compliance and pressure-distensibility curves was tested by creating non-linear regression models. An F-test was used to determine whether there was an overall effect of age on the non-linear relationship.

Afterwards mean maximal area, maximal compliance and maximal distensibility were compared between young and old horses and between investigated vessels using a multivariate ANOVA including age (young, old) and location (proximal aorta, distal aorta, caudal common carotid artery and external iliac artery) as fixed factors. Post-hoc Bonferroni correction was applied for multiple comparisons. P-values of ≤ 0.05 were considered statistically significant.

## Results

### Histological findings

**Fig 4:**
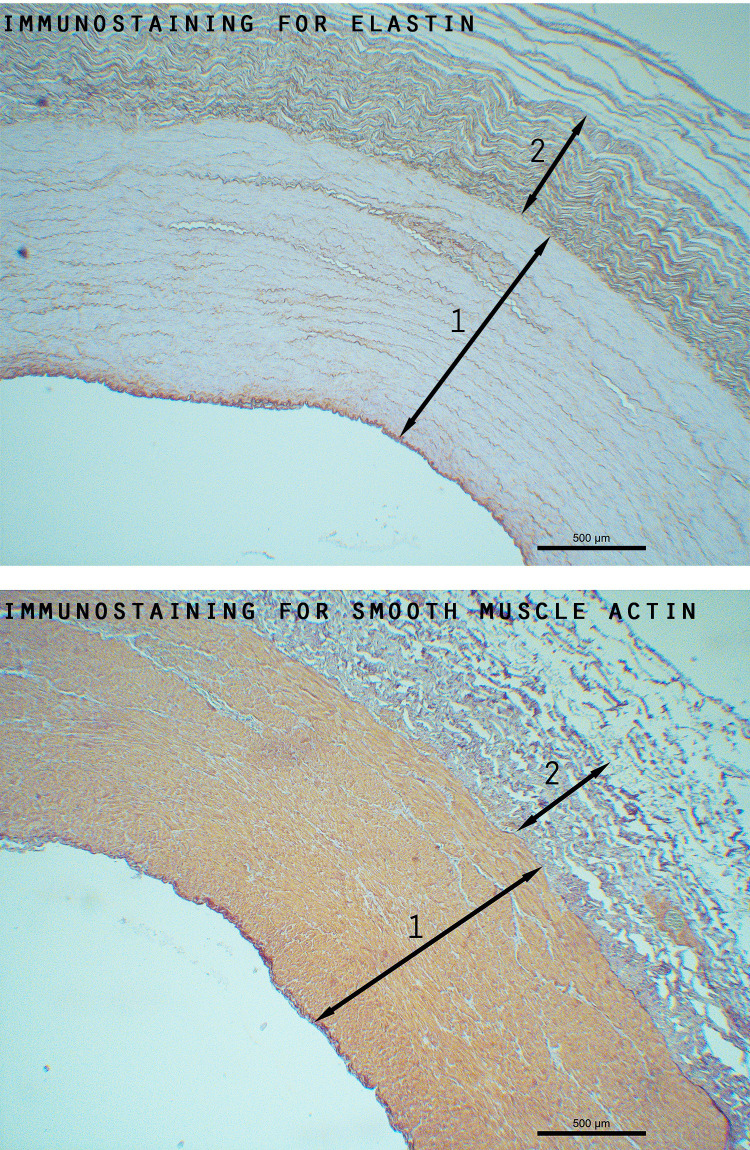
Immunostaining for elastin and smooth muscle actin of the caudal common carotid artery, showing two clearly distinguishable layers: Layer 1 (1) consisting mainly of smooth muscle cells and layer 2 (2) consisting predominantly of elastin fibres.

Arterial wall tissue was collected from 20 Warmblood horses. Six horses were categorised as young (mean age ± SD: 6±0 years) and 14 as old (mean age ± SD: 18±3 years). At the level of the proximal aorta, the tunica media consisted of one layer with an almost uniform distribution of fibres and smooth muscle cells. At the level of the distal aorta, two different layers within the tunica media could be distinguished in 40% of the horses (5 old and 3 young horses). In the innermost layer (layer 1) the smooth muscle cell content was higher than the fibre content. The outer layer (layer 2) presented a large quantity of fibres with a very limited amount of smooth muscle cells (Fig 4). The tunica media also presented these two clearly distinguishable layers in all horses at the level of the cranial and caudal common carotid, the external iliac, the femoral and the median artery.

No histological lesions were found, except for one horse (aged 18 years) in which the proximal aorta presented multifocal small mineralisations in the tunica media, close to the tunica adventitia. A Von Kossa staining indicated that the mineralisations were calcifications.

**Fig 5:**
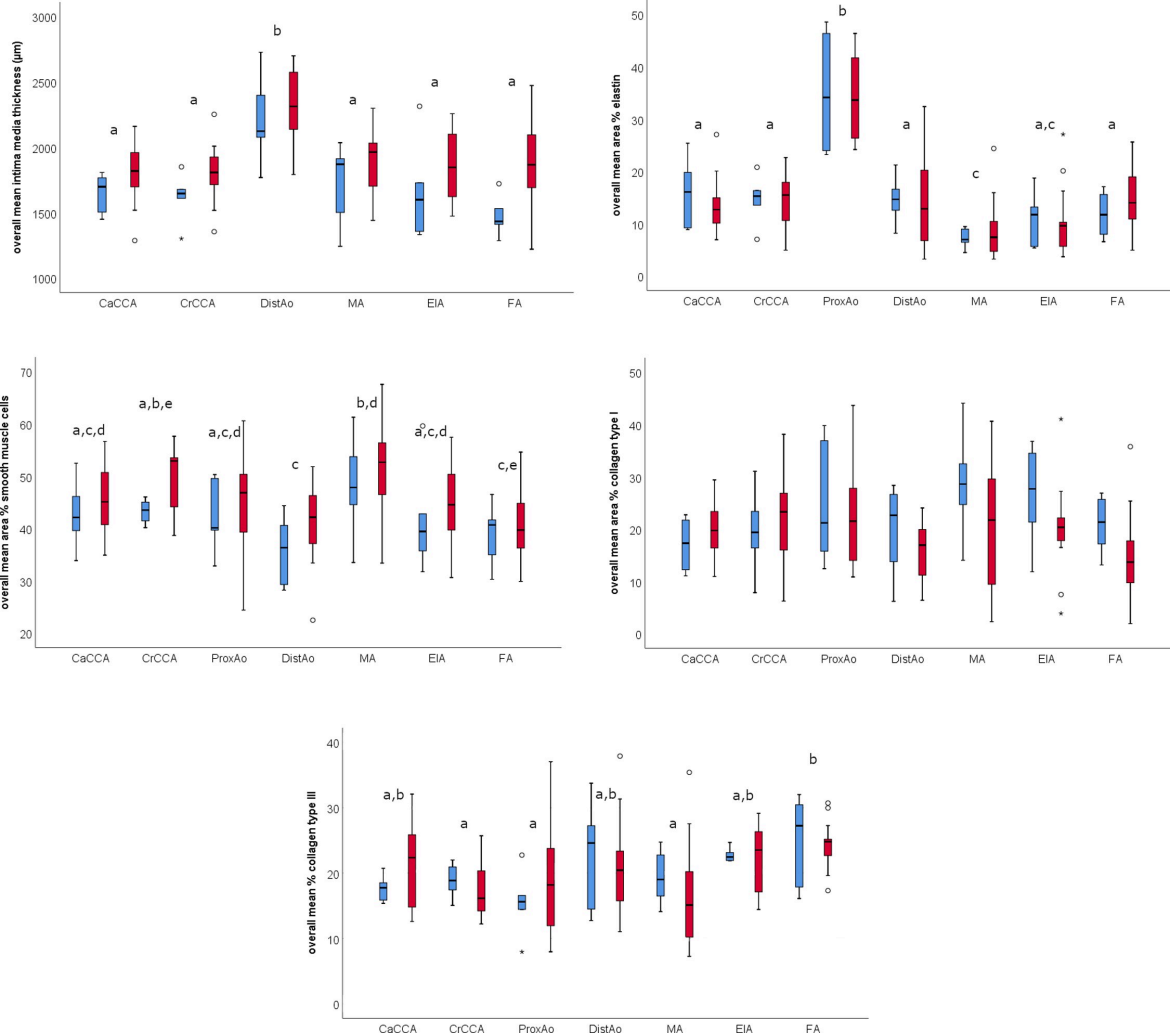
Overview of the overall mean intima media thickness, area % of elastin, area % of smooth muscle cells, area % of collagen type I and area % of collagen type III in young (blue) and old (red) horses, grouped by location. For each variable, the median and spread is indicated by a boxplot with the line in the box marking the median value, the box span indicating half of the observations and the whiskers indicating the range. Outliers (open circles) and extreme outliers (asterisks) are plotted individually at the end of the whiskers. A significant effect of age was found for the overall mean intima media thickness (p = 0.001) and the overall area % of smooth muscle actin (p = 0.027), independent from arterial location. Different characters above the boxplots indicate a significant difference between locations, independent of age. CaCCA: caudal common carotid artery; CrCCA: cranial common carotid artery; ProxAo: proximal aorta; DistAo: distal aorta; MA: median artery; EIA: external iliac artery; FA: femoral artery.

**Fig 6:**
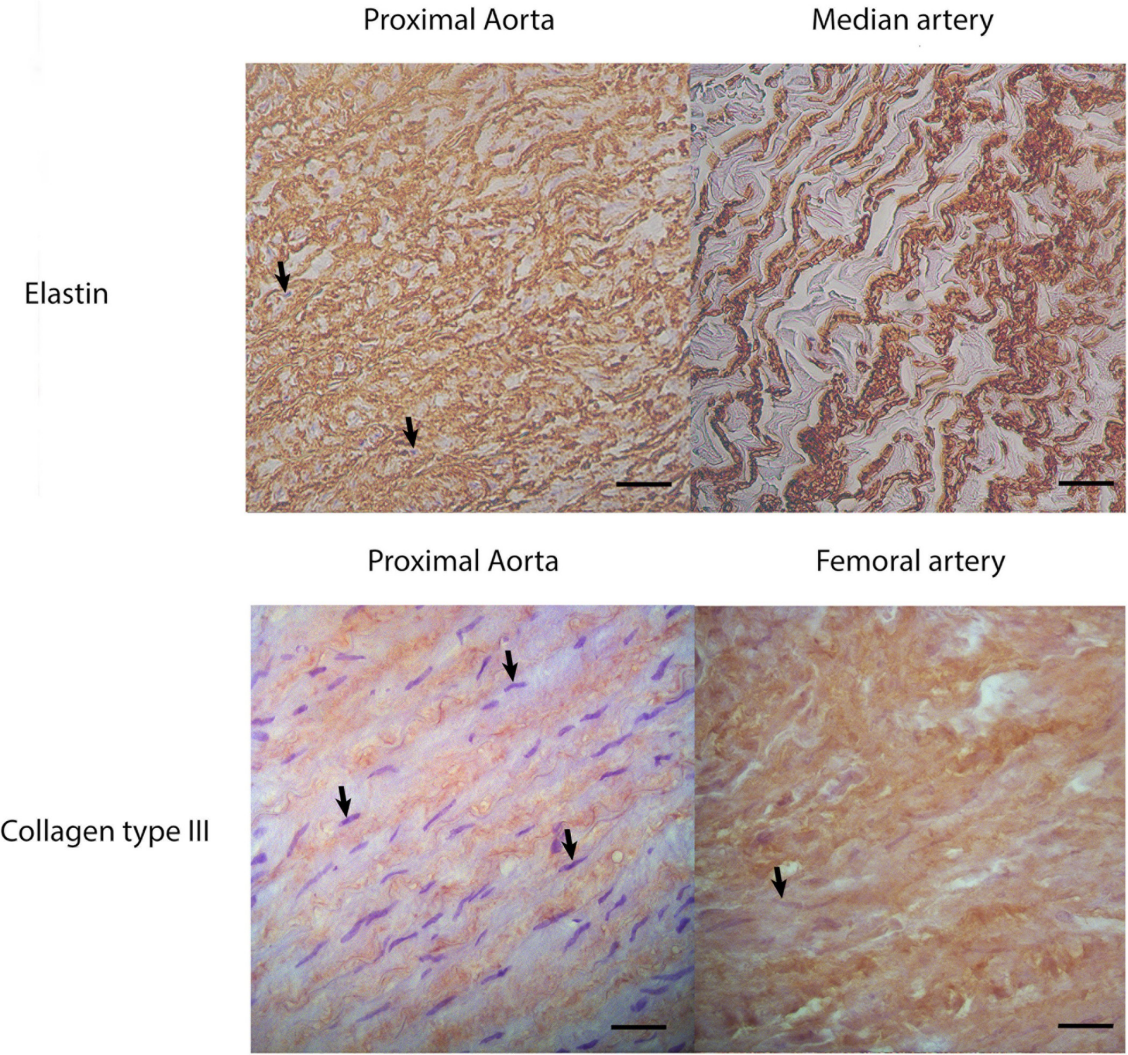
Immunohistochemistry for elastin and collagen type III (brown) of longitudinal sections through the vessel wall. The proximal aorta contains a higher area % of elastin and a lower area % of collagen type III compared to peripheral arteries. Magnification x400; bar = 250μm. Black arrows indicate nuclei of myocytes.

There was no significant interaction between location and age. There was a significant effect of location on the overall arterial wall thickness, the overall area % of elastin, the overall area % of smooth muscle actin and the overall area % of collagen type III (Table 1 and Fig 5). The distal aorta showed a clearly thicker arterial wall (2273±328 μm) compared to the other arteries. The highest overall amount of elastin was found in the proximal aorta (34±9%), whereas the median artery contained the lowest amount of elastin (8±5%). The highest overall amount of collagen type III was found in the femoral artery (25±5%) whereas the lowest overall amount of collagen type III was found in the proximal aorta (17±7%), the median artery (17±7%) and the cranial common carotid artery (18±4%) (Fig 6).

**Table 1:** Overview of overall measured intima media thickness, area % of elastin, smooth muscle actin, collagen type I and collagen type III for the caudal and cranial common carotid artery, the proximal and distal aorta, the median, the external iliac and the femoral artery in old and young horses.

		Young	Old
Thickness (μm)* (mean ± SD)	Caudal common carotid artery^a^	1654±145	1800±225
	Cranial common carotid artery^a^	1622±179	1800±231
	Distal aorta^b^	2219±361	2300±327
	Median artery^a^	1739±302	1885±265
	External iliac artery^a^	1655±360	1840±253
	Femoral artery^a^	1469±147	1870±325
% Elastin (mean ± SD)	Caudal common carotid artery^a^	16±7	14±5
	Cranial common carotid artery^a^	15±5	14±5
	Proximal aorta^b^	35±11	34±8
	Distal aorta^a^	15±4	14±9
	Median artery^c^	7±2	9±6
	External iliac artery^a,c^	11±5	11±6
	Femoral artery^a^	12±5	15±6
% Smooth muscle actin* (mean ± SD)	Caudal common carotid artery^a,c,d^	43±6	46±6
	Cranial common carotid artery^a,b,e^	43±2	50±6
	Proximal aorta^a,c,d^	42±7	45±10
	Distal aorta^c^	36±6	41±7
	Median artery^b,d^	48±9	51±10
	External iliac artery^a,c,d^	41±10	44±8
	Femoral artery^c,e^	39±6	41±7
% Collagen type I (mean ± SD)	Caudal common carotid artery	17±5	20±5
	Cranial common carotid artery	20±8	23±10
	Proximal aorta	25±11	23±11
	Distal aorta	20±9	16±6
	Median artery	29±10	20±12
	External iliac artery	27±9	20±9
	Femoral artery	21±5	15±9
% Collagen type III (mean ± SD)	Caudal common carotid artery^a,b^	18±2	21±6
	Cranial common carotid artery^a^	19±2	18±5
	Proximal aorta^a^	15±5	18±8
	Distal aorta^a,b^	23±8	21±7
	Median artery^a^	19±4	16±8
	External iliac artery^a,b^	23±1	22±5
	Femoral artery^b^	25±7	24±4

* indicates significant difference between young and old horses, independent of location; different characters in superscript indicate a significant difference between locations (level of significance p<0.05).

A significant effect of age was found for the overall arterial wall thickness (p = 0.001) and the overall area % of smooth muscle actin (p = 0.027), independent from arterial location (Table 1). The arterial wall thickness and the overall area % of smooth muscle actin were significantly higher in older horses when compared to younger animals. The increased arterial wall thickness in older horses was mainly due to an increased thickness of layer 1 (see S1 Table). No significant difference was found for the overall area % of elastin nor for the overall area % of collagen type I and III. Details can be found in Table 1.

### Inflation-extension test

**Fig 7:**
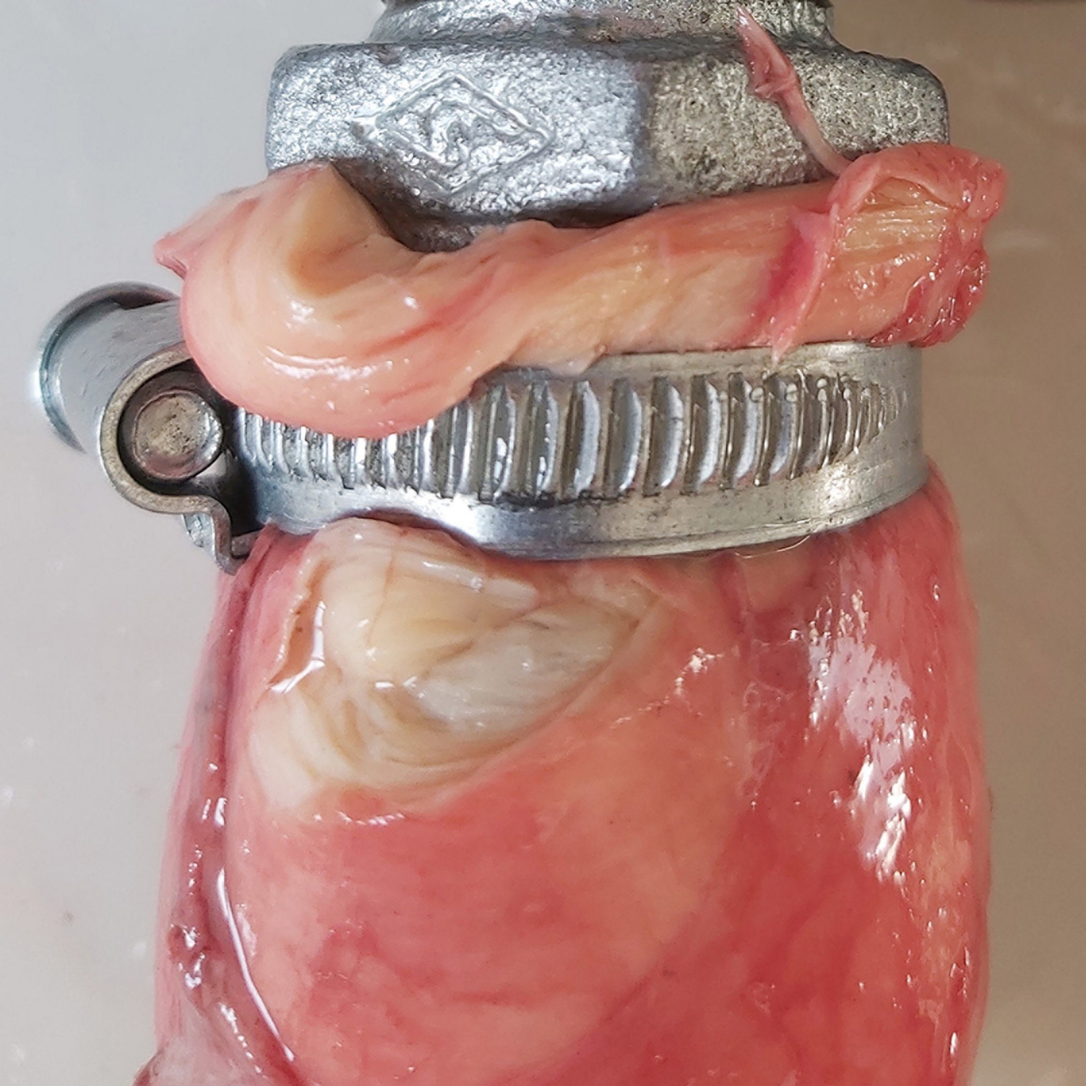
Typical cross-sectional rupture of the proximal aorta, close to the mounting side, which occurred predominantly in older horses (> 15 years) at high pressures (250–300 mmHg).

During the inflation-extension test a total of eight arteries ruptured, five of them being proximal aortas. Three proximal aortas of old horses (aged 18, 23 and 25 years) ruptured at 250 mmHg pressure while the other two, of horses aged 6 and 15 years, ruptured at 300 mmHg. Rupture always occurred in cross-sectional direction, close to the strap securing the artery to the connector (Fig 7). Two distal aortas of old horses (aged 17 and 15 years) ruptured at the bifurcation of a ligated side branch, at a pressure of 250 and 300 mmHg. One external iliac artery of an old horse (20 years) ruptured at the bifurcation of a ligated side branch at a pressure of 250 mmHg. For the external iliac artery, two samples were not tested, as they could not be made leak proof.

Results from the inflation-extension test fitted the arctangent model well, with R² values (mean ± SD) of 0.990±0.009 for the proximal aorta, 0.978±0.025 for the distal aorta, 0.982±0.247 for the carotid artery and 0.831±0.271 for the external iliac artery.

There was a significant effect of location on the mechanical properties of the arterial wall (Table 2). Largest mean maximal area was found for the proximal aorta (3202mm²) whereas the smallest area was found for the caudal common carotid artery (109mm²). The highest maximal compliance was found for the proximal aorta (17mm²/mmHg) while the lowest maximal compliance was found for the caudal common carotid artery (0.7mm²/mmHg) and the external iliac artery (0.7mm²/mmHg). The highest maximal distensibility was found for the distal aorta (0.022/mmHg) but no significant difference in maximal distensibility was found within the other three arteries.

**Table 2:** Overview of mean maximal area, compliance and distensibility of the proximal aorta, distal aorta, common carotid artery and external iliac artery.

		Young	Old
Maximal area (mm²) (mean ± SD)	Proximal aorta^a^	3313±471	3154±852
	Distal aorta^b,^	1039±198	1132±279
	Common carotid artery^c^	100±14	114±27
	External iliac artery^c^	172±64	215±53
Maximal compliance (mm²/mmHg) (mean ± SD)	Proximal aorta^a^	18±5	17±6
	Distal aorta^b^	10±4	10±5
	Common carotid artery^c^	0.7±0.3	0.8±0.3
	External iliac artery^c^	0.7±0.3	0.8±0.3
Maximal distensibility (/mmHg) (mean ± SD)	Proximal aorta^a^	0.008±0.001	0.008±0.002
	Distal aorta^b^	0.022±0.014	0.018±0.011
	Common carotid artery^a^	0.013±0.004	0.012±0.004
	External iliac artery^a^	0.006±0.003	0.006±0.004

Different characters in superscript indicate a significant difference between locations (level of significance p<0.05).

**Fig 8:**
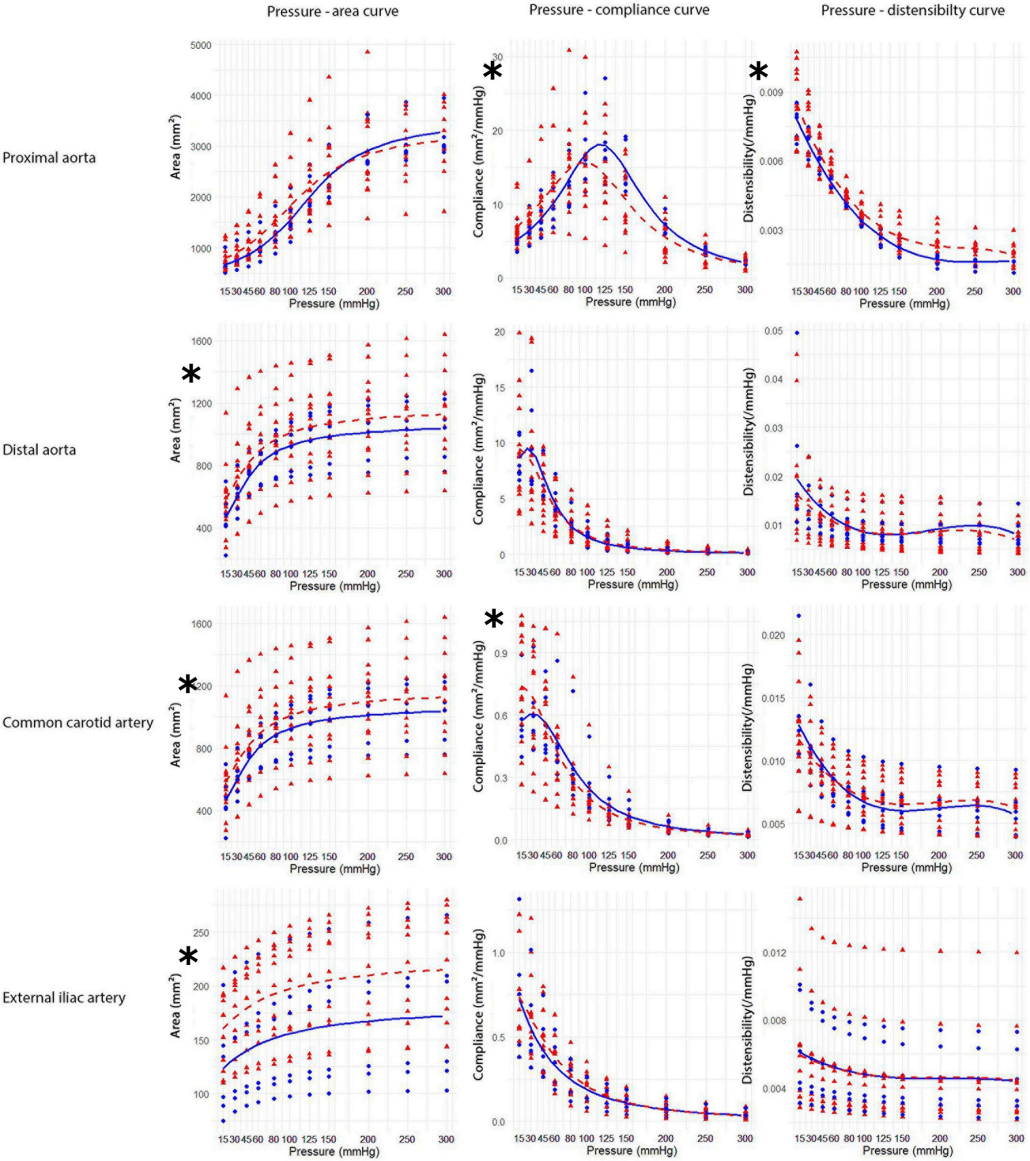
Pressure-area, pressure-compliance and pressure-distensibility curves of the proximal aorta, distal aorta, common carotid artery and external iliac artery in young (blue) and old horses (red). Dots and triangles represent individual values of young and old horses, respectively; full lines and dotted lines represent the fitted regression curves of young and old horses, respectively. Asterisks indicate a significant difference between old and young horses for the pressure-area, pressure-compliance or pressure-distensibility curves.

The pressure-area curve of the distal aorta (p = 0.048), caudal common carotid artery (p<0.001) and external iliac artery (p<0.001) were significantly affected by age, showing larger areas at the same pressures in older horses. In the proximal aorta the pressure-compliance curve showed significant age-related differences (p = 0.001) with lower maximal compliance at lower pressure in old horses. The pressure-compliance curve of the caudal common carotid artery was significantly affected by age (p = 0.038) as well. Old horses presented a higher maximal compliance at low pressures and a lower compliance at physiological pressures. The pressure-distensibility curve of the proximal aorta was significantly different between old and young horses (p<0.001), showing a slightly higher distensibility in older horses at all pressures (Fig 8). Independent from location, there was no significant influence of age on the mean maximal area, maximal compliance or maximal distensibility (Table 2).

## Discussion

The arterial wall consists of three layers, from inner to outer side: the intima, the media and the adventitia. The present histological study focussed on the tunica intima and the tunica media, which are mainly responsible for arterial distensibility and resilience. Although the adventitia does contribute to arterial stiffness and compliance [1, 21, 22], this layer was not included in the histological study because it occasionally got lost during sample processing, although it was included during the inflation-extension test.

In this study the tunica media presented two clearly distinguishable layers in 40% of the distal aortas and in all of the common carotid, median, external iliac and femoral arteries. When passing from more centrally located elastic arteries to more peripheral muscular arteries in the human cardiovascular system, the amount of elastin is known to reduce and the elastin fibres are known to become predominantly organised in the internal and external elastic lamina with a clearly defined layer of smooth muscle cells in between. The inner layer of the tunica media (layer 1) in this study matches with the clearly defined zone of smooth muscle cells, while the outer layer (layer 2) can be considered as multiple external elastic laminae. A clear internal elastic lamina was not found, although in some arteries the amount of elastin was clearly higher at the most inner side of the tunica media. As far as the authors know, this clear division of the tunica media in a pronounced layer of smooth muscle cells (layer 1) and elastic fibres (layer 2) has not been described in other species yet.

The present study showed a significant effect of location on the amount of elastin in the arterial wall. The largest amount of elastin was found in the proximal aorta (34–35%), while the lowest amount was found in the median artery and the external iliac artery (7–11%). The highest area % of collagen type III on the other hand, was found at the level of the distal aorta (25±8%), external iliac artery (23±1%) and femoral artery (25±7%). Therefore, a more elastic proximal aorta and a stiffer distal aorta and external iliac artery were hypothesized to be found in the *ex vivo* inflation-extension test. Nevertheless, the maximal distensibility, describing the maximal relative area change per mmHg, of the proximal aorta (0.008±0.001) and external iliac artery (0.006±0.003) was significantly lower compared to the distal aorta (0.022±0.14). The higher distensibility found at the level of the distal aorta compared to the external iliac artery is probably due to the significantly higher area % of elastin in the distal aorta (15±5% versus 11±5%). The low distensibility of the proximal aorta could not be explained based on current histological findings.

The present study also revealed age-related arterial wall thickening in horses, a phenomenon that has been described in human patients [3, 4]. In humans this process is known to result from smooth muscle cell phenotype switch: smooth muscle cells switch from the contractile to the secretory phenotype and migrate from the media towards the intima. In the intima they proliferate and start producing collagen and proteoglycans [10, 23–25]. In our study the increased arterial wall thickness was probably not due to smooth muscle cell phenotype switch, migration and proliferation. The overall area % of smooth muscle actin, which is only present in a very small amount in the secretory phenotype [26, 27], was increased in older horses. Moreover, no indication of an increased amount of collagen or proteoglycans in the tunica intima was present.

Characteristics of vascular aging in humans include an increase in collagen content associated with more cross-linking, a decrease in elastin content, elastin fragmentation and calcification [1, 3, 6, 28]. In the current study, elastin fragmentation in combination with calcification was found in an 18-year-old horse at the level of the proximal aorta. Age-related changes in the amount of collagen and elastin have been described in primates, birds, fish, mice, rats and dogs [29, 30] and are therefore expected to be present in horses too. In old mares, histological changes have been described in the uterine artery [31]. An increased overall arterial wall stiffness has recently been demonstrated *in vivo* in old horses [17]. Degradation of collagen type I has been described to be related to increased arterial wall stiffness in humans [32], but was not found in our study. The present study found age-related differences in the area % of smooth muscle actin but not in the amount of elastin and collagen. This could be ascribed to the small number of horses or to the fact that horses were considered old if aged over 15 years. Based on the chronological age, which refers to an animal’s numerical age in relation to the life expectancy, horses would have been considered old when aged >20 years, as the life expectancy of a horse is around 30 years of age [17, 33, 34]. Similar as in humans and rats, collagen type I and III were present in the tunica media of the equine arterial wall [35, 36]. The amount of collagen type I and III was approximately the same (Fig 5). However, in humans collagen type I accounts for only 30% and collagen type III for 70% of the total collagen in the tunica media [36]. The tunica adventitia, which consists primarily of collagen type I in human patients [35], was not investigated in our study.

Smooth muscle cells are an active component and their mechanical properties depend on the state of contraction. For our inflation-extension test, arterial tissue was frozen without cryoprotective agent, leading to smooth muscle cell death [37]. Therefore, no detectable effect of the increased amount of smooth muscle actin was expected in the inflation-extension test. Nevertheless, some changes in mechanical function were found. The pressure-compliance curve was significantly different between young and older horses for the proximal aorta and the common carotid artery. For the proximal aorta, lower compliance in old horses was found in the higher pressure ranges. The maximal compliance was lower in older horses and was reached at lower pressure. The shift of maximal compliance to lower pressures is a known phenomenon in human medicine. A remarkable difference between equine and human aortas is the higher pressure at which maximal compliance is achieved in horses (100–125 mmHg) compared to humans (0–40 mmHg) [5]. In humans, a clear difference in compliance can be found between low (0–60 mmHg) and physiological (60–200 mmHg) pressures. Maximal compliance is found at low pressure ranges and increases with increasing age, while compliance in the physiological pressure ranges decreases with increasing age [5, 38, 39]. This phenomenon of maximal compliance in the low pressure range was not found in the proximal aorta of horses. For the common carotid artery, pressure-compliance curves behaved as expected: higher maximal compliance at low pressure (15-45mmHg) and lower compliance at physiological pressures were found in older horses compared to younger horses. No significant age difference was found in the pressure-distensibility curve, except for the proximal aorta. This location showed slightly higher distensibility in older horses at all pressures, in contrast to human studies [5, 40, 41].

Tissue storage might have influenced the mechanical properties of the arterial wall. Storage at -20 or -80°C is preferred over storage at 4°C, but even then biomechanical changes may occur [42] due to a loss of collagen. A normal stress-strain curve shows a knee-point, between the initial and the stiff slope. This knee-point is the point at which collagen fibres are being recruited to help bear the stress load, up till then carried by elastin. In thawed tissue, due to a loss of total collagen, the knee-point will shift to a higher strain. The amount of collagen loss did not increase with increasing storage time [42]. However, effects are probably small as Stemper et al. (2017) [43] found no difference in ultimate tensile strength between fresh porcine arterial tissue and tissue frozen at -20 and -80°C for 3 months. The elastin network, the most important in determining tissue compliance, was not affected by freezing in any of the studies. On the contrary, Venkatasubramanian et al. (2006) [44] found clear differences between fresh and frozen arterial tissue in the lower strain region, indicating that freezing does also affect elastin. The preservation of smooth muscle cells during freezing depends on the freezing protocol and the applied medium. Without cryoprotective agent, as in our study, freezing will lead to smooth muscle cell death [37]. Cryopreservation in foetal calf serum, containing 1.8 M DMSO as cryoprotecting agent, could have been applied as it has been shown to preserve maximal contractile response after freezing. The serum is responsible for the maintenance of endothelial function, while DMSO is necessary for the preservation of the smooth muscle cells [38]. Preservation of smooth muscle cells would lead to a horizontal shift of the pressure at which the compliance is maximal as shown in human subjects [5].

Another important issue is the time between death and the collection and storage of the tissue. Post mortem changes start immediately after death and the rate at which they occur is determined by several factors such as ambient temperature, content of body fat and injury. Therefore samples were collected as soon as possible after death and always within 12h after death, to avoid the peak activity of autolysis, which occurs around 24h after death [45].

*In vivo*, arteries are longitudinally stretched. Pressure-inflation tests should therefore be carried out at *in vivo* length, which normally corresponds to 1.6 times the unloaded length in humans and mice [46]. *In vivo* lengths were not determined in our study. However, the degree to which the vessel is pre-stretched does influence the inflation curve. In order to approach the *in vivo* situation and allow for better comparison between old and young horses, samples were investigated at a fixed length: the length of the vessel at 120 mmHg, the mean arterial pressure in horses [47]. It was visually observed that such pressurisation induced an elongation in the order of 1.5–2 times the unloaded length.

Arteries were pressurised up to 300 mmHg, as horses may reach such high pressures during exercise [48]. Rupture occurred in a minority of arteries (8/78) at high pressures (between 250–300 mmHg) and mostly occurred in older horses (7/8 in old horses). Rupture occurred most frequently just underneath the mounting place. As mounting was the same in old and young horses, results indicate a predisposition of older horses to arterial rupture. The proximal aorta with calcifications in the tunica media did not rupture during the inflation-extension test. A limitation of our study is that the horses were not examined ante-mortem to confirm that they were cardiovascular healthy.

In conclusion, clear differences in histological and biomechanical properties of the arterial wall were found between the elastic central arteries and the more muscular, peripheral arteries. The proximal aorta contained the largest area % of elastin, while the distal aorta, external iliac and femoral artery contained the highest area % of collagen type III. The highest distensibility was found for the distal aorta, and not for the proximal aorta as expected. Histological comparison of the arterial wall of old and young horses revealed a thicker arterial wall in old horses and a higher area % of smooth muscle actin. An *ex vivo* inflation-extension test revealed larger cross-sectional vessel areas in old horses compared to young horses at the same pressures. Lower maximal compliance at lower pressures in old horses was found for the proximal aorta, while higher maximal compliance at low pressures in combination with lower compliance at physiological pressures was found at the level of the common carotid artery.

## Supporting information

S1 Table Measured thickness, area % of elastin, smooth muscle cells, collagen type I and collagen type III within layer 1 and layer 2 for the caudal and cranial common carotid artery, distal aorta, median artery, external iliac artery and femoral artery in young and old horses. (DOCX)

S1 File Dataset histological findings. (PDF)

S2 File Dataset inflation-extension test. (PDF)
