# Supplementary material for: Histological and biomechanical properties of systemic arteries in young and old Warmblood horses
Source: PLoS One. 2021 Jul 12;16(7):e0253730. doi: 10.1371/journal.pone.0253730 (PMC8274928; doi:10.1371/journal.pone.0253730)
Supplement: S1 Table — (DOCX) [file pone.0253730.s001.docx]

**S1 Table. Measured thickness, area % of elastin, smooth muscle cells, collagen type I and collagen type III within layer 1 and layer 2 for the caudal and cranial common carotid artery, distal aorta, median artery, external iliac artery and femoral artery in young and old horses.**

|  |  | Layer 1 | | Layer 2 | |
| --- | --- | --- | --- | --- | --- |
|  |  | Young | Old | Young | Old |
| Thickness (µm) | Caudal common carotid artery | 1209±153 | 1554±647 | 441±63 | 424±88 |
|  | Cranial common carotid artery | 1179±152 | 1405±246 | 436±70 | 401±68 |
|  | Distal aorta | 1673±434 | 1972±380 | 408±3 | 429±47 |
|  | Median artery | 1367±253 | 1549±240 | 381±155 | 338±107 |
|  | External iliac artery | 1275±253 | 1571±213 | 353±84 | 307±74 |
|  | Femoral artery | 1127±162 | 1701±579 | 344±54 | 361±83 |
| % Elastin | Caudal common carotid artery | 14±6 | 10±4 | 21±10 | 23±8 |
|  | Cranial common carotid artery | 12±4 | 12±5 | 22±9 | 23±8 |
|  | Distal aorta | 17±2 | 17±13 | 29±12 | 23±9 |
|  | Median artery | 1±1 | 4±6 | 30±6 | 28±6 |
|  | External iliac artery | 8±5 | 8±6 | 24±6 | 24±7 |
|  | Femoral artery | 7±4 | 12±5 | 29±11 | 28±8 |
| % Smooth muscle actin | Caudal common carotid artery | 57±7 | 60±7 | 3±3 | 1±1 |
|  | Cranial common carotid artery | 59±4 | 63±7 | 1±1 | 2±4 |
|  | Distal aorta | 44±13 | 46±11 | 2±2 | 2±2 |
|  | Median artery | 61±9 | 62±11 | 2±2 | 2±2 |
|  | External iliac artery | 53±12 | 52±10 | 1±1 | 4±3 |
|  | Femoral artery | 51±6 | 50±7 | 1±1 | 2±3 |
| % Collagen type I | Caudal common carotid artery | 23±7 | 25±6 | 2±2 | 1±1 |
|  | Cranial common carotid artery | 23±7 | 25±3 | 2±1 | 3±4 |
|  | Distal aorta | 16±11 | 20±6 | 0±0 | 2±2 |
|  | Median artery | 36±11 | 24±14 | 2±2 | 2±2 |
|  | External iliac artery | 34±11 | 24±10 | 1±1 | 2±1 |
|  | Femoral artery | 34±11 | 23±10 | 1±1 | 2±1 |
| % Collagen type III | Caudal common carotid artery | 14±2 | 20±7 | 29±5 | 27±6 |
|  | Cranial common carotid artery | 15±3 | 15±5 | 29±5 | 27±6 |
|  | Distal aorta | 28±4 | 26±10 | 42±5 | 27±10 |
|  | Median artery | 17±5 | 14±8 | 29±8 | 28±9 |
|  | External iliac artery | 20±2 | 22±6 | 32±5 | 26±4 |
|  | Femoral artery | 25±10 | 23±5 | 28±6 | 27±7 |
