# Supplementary material for: Histological and biomechanical properties of systemic arteries in young and old Warmblood horses
Source: PLoS One. 2021 Jul 12;16(7):e0253730. doi: 10.1371/journal.pone.0253730 (PMC8274928; doi:10.1371/journal.pone.0253730)
Supplement: S1 File — (PDF) [file pone.0253730.s002.pdf]

Samplenr	Horse	Location	Age	Thickness	Elastin	SMC	Collagen3	Collagen1
1	1	1	2	1286,8	27,05325	39,26327	26,20999	10,95178
2	1	2	2	1354,6	22,65732	55,202	18,98118	15,46842
3	1	3	2		45,328	60,5824	37,0414	27,2436
4	1	4	2	1805,2	25,77839	39,04418	37,89647	11,14017
5	1	5	2	1441,4	15,91837	47,22772	35,40419	2,594643
6	1	6	2	1473,8	27,08944	47,3159	19,20864	7,496298
7	1	7	2	1220,8	19,61399	34,52747	29,95735	11,72238
8	2	1	2	1820,6	14,47043	46,20325	23,97953	18,03983
9	2	2	2					
10	2	3	2		36,1304	46,917	25,5768	10,8728
11	2	4	2		23,1308	51,8232	22,8236	16,6872
12	2	5	2	1957	10,81487	35,66196	27,54271	9,442378
13	2	6	2	2100	8,584497	57,44609	26,33525	17,80388
14	2	7	2	1846,6	18,96223	39,54522	30,71439	3,305187
15	3	1	2	1961,8	12,71973	39,0292	25,84455	15,00843
16	3	2	2	1805,8	12,3114	53,0707	16,13815	23,3024
17	3	3	2		41,705	32,5182	12,5324	14,0238
18	3	4	2		20,2286	39,8096	21,133	22,3076
19	3	5	2	1756,6	24,38239	33,37591	20,21053	11,54898
20	3	6	2	1528,746	20,10404	44,37719	25,53611	19,3465
21	3	7	2	2186,8	13,65337	49,0582	25,1898	14,03597
23	4	1	2	1714,4	11,14586	52,91686	18,75548	29,426
24	4	2	2	1714,4	11,14888	52,91736	20,33592	38,13168
25	4	3	2		42,4552	46,5236	21,1626	27,8196
26	4	4	2	2641,2	32,39978	36,37753	28,30306	18,25761
27	4	5	2	1833,4	7,56549	56,03324	22,59795	19,66851
28	4	6	2	1848,2	10,02533	44,70034	27,3946	21,92722
29	4	7	2	1819,2	9,194656	36,24924	24,71804	25,38214
30	5	1	1	1696,6	19,81698	39,60535	20,72865	14,19552
31	5	2	1	1631,6	16,16441	40,15974	19,15448	23,39356
32	5	3	1		35,023	50,2966	15,475	36,919
33	5	4	1	2397,8	21,23756	29,28775	27,24257	19,11752
34	5	5	1	1242,8	6,760187	44,55322	22,76639	29,19073
35	5	6	1	1728	18,72899	36,85816	24,68975	21,34563
36	5	7	1	1721,6	17,07673	39,76621	27,90895	21,99755
37	6	1	2	1786,4	13,86124	34,90399	24,38865	23,38644
38	6	2	2	1724,4	15,89185	44,14688	14,3441	24,89089
39	6	3	2		46,3808	39,9484	12,254	11,9226
40	6	4	2	2574	10,9674	37,1102	11,0268	11,2116
41	6	5	2	2259	8,838893	50,12836	15,1962	23,81642
42	6	6	2	1751,8	9,969931	30,62661	17,13285	3,873187
43	6	7	2	1648,4	14,33874	29,87335	23,17228	9,748761
44	7	1	1	1767	19,20161	46,11943	15,35856	21,71705
45	7	2	1	1678	16,3323	41,4578	18,55913	31,06359
46	7	3	1		23,9708	49,5472	15,7542	39,7952
47	7	4	1		12,5692	28,209	14,4844	26,6424
48	7	5	1	1896,2	4,48211	45,05216	16,49495	28,04403
49	7	6	1	2313,8	5,345051	31,73808	21,92965	36,80314
50	7	7	1	1410,4	7,981225	41,64022	17,87628	26,89498

51	8	1	1	1503,8	9,204653	33,8539	15,87351	20,43077
52	8	2	1	1301,2	13,53961	45,02892	15,047	20,61882
53	8	3	1		33,2672	32,8482	16,5706	21,7144
54	8	4	1	2727,2	8,168	34,0794	12,7332	26,1794
55	8	5	1	1912,6	7,155439	33,50104	14,0923	14,04236
56	8	6	1	1357,2	5,637838	35,71132	21,88337	25,35131
57	8	7	1	1416,2	6,554345	30,27241	26,58918	25,69718
58	9	1	2	2050	10,83463	42,33334	20,75297	20,53159
59	9	2	2	1807,6	7,587316	44,78289	16,62048	9,913557
60	9	3	2		24,1898	46,7316	27,0728	15,6038
61	9	4	2	2170	17,9464	47,339	15,7506	6,3916
62	9	5	2	1641,2	4,705889	46,4863	16,3677	9,145268
63	9	6	2	2122,4	5,662642	36,04236	16,04362	23,08337
64	9	7	2	2095,6	11,27001	38,34928	20,31841	17,77677
65	10	1	2	1932,2	10,0935	40,7469	14,7918	16,39245
66	10	2	2	1896,6	4,928572	38,66014	13,56729	17,1187
67	10	3	2		26,363	35,5768	7,964	20,5256
68	10	4	2	2264	6,024069	22,46484	13,96887	23,58933
69	10	5	2	2000	4,89398	38,15662	11,9427	28,81108
70	10	6	2	1764,2	10,12689	45,09172	29,13891	20,15239
71	10	7	2	1403,8	4,926594	44,81757	27,25881	23,36377
72	11	1	2	1816	9,275332	42,14745	32,07549	24,56507
73	11	2	2	1968,8	7,862683	41,50135	15,3931	16,00038
74	11	3	2		34,064	24,3866		22,5422
75	11	4	2		3,2208	42,0778	11,5848	24,0584
76	11	5	2	1441,2	3,230149	53,35503	8,024513	40,63368
77	11	6	2	1850,75	3,692064	39,71876	23,19436	41,04633
78	11	7	2	2472,6	10,88852	39,89212	22,67621	35,77509
79	12	1	2	2160	14,95034	50,05216	14,74986	20,47487
80	12	2	2	1925,4	20,70204	53,36772	12,21484	37,5485
81	12	3	2		27,5752	56,0708	10,7162	43,663
82	12	4	2	2699,8	9,792847	44,74441	19,49875	17,21306
83	12	5	2	1970,2	5,360587	53,20801	8,169647	14,83236
84	12	6	2	1622,8	5,474201	55,28856	23,85651	19,09958
85	12	7	2	1828	18,38823	49,04582	25,04037	17,0347
86	13	1	2	1876	6,931645	56,64997	12,60202	27,34528
87	13	2	2	2008,2	19,59452	53,54303	21,91624	26,87909
88	13	3	2		25,9044	50,349	18,1838	26,9034
89	13	4	2		5,3444	45,6906	19,7264	18,3608
90	13	5	2	1702,6	3,696906	65,73944	15,01064	29,60507
91	13	6	2	1530	5,267014	50,34975	22,90175	22,1382
92	13	7	2	1691,2	12,83537	39,97186	25,16573	14,35553
93	14	1	1	1449,4	8,880122	42,04599	18,51249	12,23137
94	14	2	1	1609,4	7,009069	44,62104	20,92934	16,4072
95	14	3	1		23,1984	39,8022	7,9418	15,8026
96	14	4	1	2077,2	12,8132	44,3652	23,187	13,7814
97	14	5	1	1843,6	6,451366	61,27673	24,73866	44,09719
98	14	6	1	1332,2	13,19391	42,80464	23,13367	34,5214
99	14	7	1	1451	8,762215	34,96083	32,01495	20,69933
100	15	1	1	1808,2	12,93775	52,49606	17,62413	11,08335

101	15	2	1	1850,6	14,391	46,0212	17,43416	7,85887
102	15	3	1		48,571	40,4242	22,7458	12,4418
103	15	4	1	1768,8	16,50681	40,58187	33,74768	6,198649
104	15	5	1	2035,2	9,454468	53,73085	17,12949	24,71383
105	15	6	1	1669	12,97369	41,99624	22,46837	11,8476
106	15	7	1	1285,6	14,65626	46,50436	30,44969	13,15228
107	16	1	1	1700,8	25,38358	42,18809	17,87705	22,74165
108	16	2	1	1662,2	20,78683	42,41902	21,99231	18,15883
109	16	3	1		46,3728	39,6738	14,4388	20,6686
110	16	4	1	2123,4	16,5886	38,5872	26,0106	28,3716
111	16	5	1	1501,8	8,960495	50,63683	20,90595	32,51508
112	16	6	1	1529	10,4917	59,6001	22,44413	30,07772
113	16	7	1	1531	15,59049	41,59811	16,1146	17,17311
114	17	1	2	1518,6	19,05876	43,98616	26,08626	21,09962
115	17	2	2	1585	15,46168	52,02514	25,63763	26,83167
116	17	3	2		36,8992	39,303	10,2846	29,0008
117	17	4	2	2550,4	6,76461	33,39704	19,32499	12,63788
118	17	5	2	2032	8,490268	52,13052	15,27372	29,40303
119	17	6	2	1872	9,226076	43,40241	24,78007	21,49538
120	17	7	2	1935,8	21,36453	32,07841	23,85637	12,14022
121	18	1	2	1625,6	6,936807	50,70758	24,0287	11,51235
122	18	2	2	1517,8	16,64754	42,00989	25,70533	17,89268
123	18	3	2		24,3536	47,1724	23,7608	14,5564
124	18	4	2	1792	8,6996	42,199	21,937	7,9402
125	18	5	2	1975,4	3,420771	56,35438	7,254679	31,36417
126	18	6	2	1844,6	10,27713	33,09466	27,96187	16,50431
127	18	7	2	1887,4	10,64659	37,34078	24,88799	13,38838
128	19	1	2	1959,4	12,68271	51,02035	13,04565	18,60505
129	19	2	2	2252,2	10,61058	57,6333	14,21009	6,240459
130	19	3	2		33,1942	55,7166	11,9466	40,8402
131	19	4	2	2361,8	16,46435	46,28585	31,3373	14,94392
132	19	5	2	2086,6	10,449	57,34262	11,58287	2,296397
133	19	6	2	2257,4	16,25482	51,8347	15,88068	20,58587
134	19	7	2	1912,4	25,62322	43,12898	17,31524	9,154133
135	20	1	2	1697,4	20,06648	49,78273	19,56738	19,0095
136	20	2	2	1845	17,91874	54,70563	14,11485	35,44936
137	20	3	2		28,0636	49,9982	18,45	11,3932
138	20	4	2	2137,8	14,7168	48,1344	23,351	19,9438
139	20	5	2	2298,8	7,252827	67,55815	10,20018	31,34287
140	20	6	2	2188	6,839092	41,16339	14,42888	27,24758
141	20	7	2	2230,2	17,45457	54,61607	19,61574	1,924893
