# Supplementary material for: Histological and biomechanical properties of systemic arteries in young and old Warmblood horses
Source: PLoS One. 2021 Jul 12;16(7):e0253730. doi: 10.1371/journal.pone.0253730 (PMC8274928; doi:10.1371/journal.pone.0253730)
Supplement: S2 File — (PDF) [file pone.0253730.s003.pdf]

Horse	Age	Vessel	Amax	P0	P1	age	Area	Compliance	Distentibility	MaxArea	MaxCompliance	MaxDistentibility
1	2	1	2645,76	81,5761	82,63	2				2132,698	10,188	0,008
1	2	2	929,595	0,184108	24,3966	2	244989	286,926	170,454	905,570	8,861	0,014
1	2	3	122,823	17,9234	40,8789	2	30093,2	58,893	41,775	117,196	0,952	0,016
1	2	4	199,297	-51,761	61,0005	2	50767,43	36,384	27,379	188,404	0,473	0,003
2	2	1	3838,53	109,724	90,3524	2	661192	2373,456	1982,140	3296,857	13,368	0,007
2	2	2	1240,51	31,3729	35,8094	2	298870,5	739,135	540,084	1188,181	11,011	0,020
2	2	3	174,86	49,7345	54,3393	2	38484,96	107,722	85,888	162,960	1,017	0,013
2	2	4	239,063	-41,7793	67,9299	2	59694,63	52,043	39,253	224,133	0,659	0,004
3	2	1	4117,62	142,774	77,3831	2	625715,7	2799,671	2342,948	3518,155	16,791	0,006
3	2	2	1530,4	45,2548	66,999	2	332668,1	850,798	674,869	1405,117	7,271	0,011
3	2	3	120,098	10,2869	56,3346	2	28778,43	49,854	36,844	112,756	0,674	0,011
3	2	4	191,005	-13,668	65,1741	2	46548,91	58,325	43,389	178,549	0,782	0,006
4	2	1	3221,4	89,7948	61,3542	2	615566,4	2242,068	1857,900	2930,197	16,298	0,010
4	2	2	1066,36	30,6996	84,3876	2	232407,4	494,957	391,110	963,285	4,022	0,008
4	2	3	121,096	21,9086	35,749	2	29821,19	63,191	44,374	116,168	1,039	0,020
4	2	4	155,001	-47,8832	81,8663	2	38013,51	34,012	26,213	143,598	0,379	0,003
5	1	1	3371,51	114,026	64,1072	1	584483,8	2417,513	2007,250	3015,261	16,264	0,008
5	1	2	898,385	19,2647	42,5997	1	218593,6	436,929	311,145	855,320	6,646	0,016
5	1	3	99,9703	24,0845	33,2297	1	24667,5	54,773	38,468	96,156	0,928	0,021
5	1	4	127,237	-19,9914	48,6138	1	32307,29	32,618	23,353	121,131	0,549	0,006
6	2	1	4259,59	66,7381	65,1389	2				3643,143	20,595	0,010
6	2	2	1763,63	23,8399	62,8903	2	406135,5	839,030	639,066	1637,930	8,842	0,011
6	2	3	169,628	10,2434	49,7044	2	41261,76	71,002	51,234	160,455	1,076	0,012
6	2	4	299,818	-47,4507	74,3332	2	74414,98	63,580	48,483	279,704	0,753	0,003
7	1	1	3355,75	125,3	83,675	1	547560,5	2189,861	1831,680	2878,623	12,766	0,007
7	1	2	773,921	26,0033	13,7812	1	201513,5	527,902	309,218	761,541	16,489	0,049
7	1	3	134,542	57,5314	49,5666	1	29216,28	89,468	72,461	125,906	0,862	0,013
7	1	4	140,093	-39,703	79,0977	1	34252,78	33,098	25,366	129,892	0,381	0,004
8	1	1	4474,21	102,793	75,7074	1	801355,3	2955,480	2455,474	3952,177	18,786	0,008
8	1	2	1329,29	34,7548	44,3066	1	311083,4	775,227	586,298	1259,258	9,441	0,016
8	1	3	98,8017	30,0049	52,8008	1	22938,92	52,286	39,907	92,728	0,596	0,014
8	1	4	282,951	-35,3071	64,4958	1	70676,65	65,258	48,761	265,836	0,868	0,004

9	2	1	1964,69	60,5741	102,376	2	388119,7	995,412	814,584	1711,998	6,108	0,007
9	2	2	1103,77	13,8476	47,2159	2	268342,8	489,071	350,056	1046,316	7,437	0,013
9	2	3	118,701	21,4371	37,4732	2	29129,12	60,964	43,108	113,649	0,979	0,019
10	2	1	4137,49	115,799	54,4284	2	718130,7	3141,007	2604,444	3759,104	23,525	0,008
10	2	2	1325,25	16,3443	42,9547	2	324015,3	616,091	435,437	1261,851	9,811	0,015
10	2	3	105,478	16,4321	48,9427	2	25416,72	48,274	35,276	99,740	0,685	0,013
10	2	4	292,271	-56,1895	131,497	2	67259,52	67,354	54,140	259,369	0,547	0,003
11	2	1	5715,35	87,335	58,149	2				4848,544	30,796	0,010
11	2	2	1534,69	-0,61911	16,6593	2	414137,9	391,616	208,615	1507,646	15,606	0,014
11	2	3	137,645	5,80481	40,3509	2	34494,33	53,563	36,826	131,673	1,032	0,013
11	2	4	293,593	-13,4373	63,6425	2	71746,15	89,548	66,300	274,872	1,224	0,007
12	2	1	2667,17	95,3388	71,4866	2				2299,589	11,826	0,009
12	2	2	1012,98	25,4948	15,6428	2				990,550	19,034	0,045
12	2	3	145,392	13,2161	51,7194	2	35018,9	63,258	46,243	137,135	0,894	0,012
12	2	4	257,372	-4,03016	31,7146	2	66823,67		49,968	248,857	1,201	0,011
13	2	1	3457,4	140,111	77,9719	2	531545,6	2342,729	1959,905	2958,060	13,891	0,006
13	2	2	1277,73	15,4343	70,6244	2	294496,7	545,566	416,507	1178,790	5,759	0,009
13	2	3	101,493	-13,639	77,419	2	24166,85	31,699	24,208	93,675	0,367	0,006
13	2	4	278,298	-21,1834	54,9315	2	69829,49	73,282	53,243	263,293	1,125	0,006
14	1	1	3351,34	115,987	65,3843	1	575638,3	2391,781	1985,938	2987,136	16,011	0,008
14	1	2	1100,94	15,4528	48,3459	1	266152	497,871	359,163	1041,962	7,248	0,013
14	1	3	92,2897	13,5731	50,5238	1	22275,34	40,451	29,584	87,161	0,581	0,012
14	2	4	149,945	-27,7074	43,5954	2	38750,66	32,070	22,817	143,633	0,559	0,005
15	1	1	4358,24	116,413	49,8784	1				3862,502	27,013	0,009
15	1	2	1301,67	8,91133	55,4693	1	313243,8	531,251	388,308	1223,651	7,381	0,011
15	1	3	105,241	19,316	63,298	1	24436,04	47,742	36,374	97,811	0,527	0,010
16	1	1	3578,65	139,17	58,6372	1	556223,6	2688,987	2214,439	3180,398	18,786	0,007
16	1	2	1128,89	27,6613	27,6342	1	280531,1	681,635	468,831	1092,552	12,911	0,026
16	1	3	107,492	39,0763	78,2316	1	23242,71	54,260	43,242	97,525	0,435	0,009
16	1	4	233,668	-40,5701	142,454	1	52144,81	59,928	48,525	204,202	0,453	0,003
17	2	1	2702,67	84,4942	52,1627	2	533485,9	1959,173	1625,308	2498,369	16,371	0,011
17	2	2	663,708	25,6655	36,1041	2	161955,9	365,894	259,896	636,063	5,768	0,020
17	2	3	101,247	13,0808	68,4278	2	23538,57	42,429	32,386	93,702	0,471	0,009

17	1	4	106,365	-6,433	29,2816	1	27865,15	29,182	18,764	103,140	0,753	0,010
18	2	1	4300,66	106,573	64,602	2	768029,4	3041,723	2526,488	3859,397	20,973	0,008
18	2	2	1317,17	22,1727	18,3021	2				1274,170	19,858	0,040
18	2	3	92,3215	9,54585	52,2194	2	22354,78	38,148	27,874	87,094	0,557	0,011
18	1	4	220,46	-2,05098	47,1716	1	54978,44	75,790	53,433	209,589	1,316	0,010
19	2	1	2943,62	93,5976	55,0649	2	559226,8	2144,588	1782,504	2699,337	16,789	0,010
19	2	2	794,245	-15,7864	51,4093	2	199584,9	222,272	159,111	753,445	3,620	0,007
19	2	3	72,5606	-12,7101	77,4111	2	17256,75	22,892	17,527	66,956	0,264	0,006
19	2	4	167,594	1,84664	10,6818	2	45996,88		17,443	165,684	0,628	0,015
20	2	1	4658,76	106,866	90,5129	2	810575,4	2864,704	2390,307	4008,855	16,290	0,007
20	2	2	1137,19	13,2967	27,5981	2	291287	515,662	321,121	1102,453	13,066	0,022
20	2	3	103,998	2,48502	50,6152	2	25563,04	38,730	27,886	98,420	0,616	0,010
20	2	4										
